# Synergistic Effects of Aluminum Diethylphosphinate and Melamine on Improving the Flame Retardancy of Phenolic Resin

**DOI:** 10.3390/ma13010158

**Published:** 2019-12-31

**Authors:** Ru Zhou, Wenjuan Li, Jingjing Mu, Yanming Ding, Juncheng Jiang

**Affiliations:** 1 Jiangsu Key Laboratory of Urban and Industrial Safety, College of Safety Science and Engineering, Nanjing Tech University, Nanjing 211816, China; 201761100038@njtech.edu.cn (W.L.); 201861201043@njtech.edu.cn (J.M.); jcjiang@njtech.edu.cn (J.J.); 2 Faculty of Engineering, China University of Geosciences, Wuhan 430074, China; dingym@cug.edu.cn

**Keywords:** phenolic resin, aluminum diethylphosphinate, melamine, flame retardancy

## Abstract

A series of novel flame retardants (aluminum diethylphosphinate and melamine) were used to improve the fire performance of phenolic resin. Fourier transform infrared spectroscopy (FTIR) was used to characterize the modification results. Thermo-gravimetric analysis (TGA) was used to study the thermal decomposition of phenolic resin system, and the flame retardancy of phenolic resin system was tested by vertical combustion test (UL-94) and limiting oxygen index (LOI). The combustion properties of modified phenolic resin were further tested with a cone calorimeter(CCT). Finally, the structure of carbon residue layer was measured by scanning electron microscopy (SEM). The results show that with the introduction of 10 wt % aluminum diethylphosphinate in phenolic resin, the LOI reaches 33.1%, residual carbon content increase to 55%. The heat release rate (HRR) decreased to 245.6 kW/m^2^, and the total heat release (THR) decreased to 58.6 MJ/m^2^. By adding 10 wt % aluminum diethylphosphinate and 3 wt % melamine, the flame retardancy of the modified resin can pass UL-94 V-0 flame retardant grade, LOI reaches 34.6%, residual carbon content increase to 59.5%. The HRR decreases to 196.2 kW/m^2^ at 196 s, relatively pure phenolic resin decreased by 35.5%, and THR decreased to 51 MJ/m^2^. Compared with pure phenolic resin, the heat release rate and total heat release of modified phenolic resin decreased significantly. This suggests that aluminum diethylphosphinate and melamine play a nitrogen-phosphorus synergistic effect in the phenolic resin, which improves the thermal stability and flame retardancy of the phenolic resin.

## 1. Introduction

With the development of world’s social economy, high-rise buildings and skyscrapers have occupied a certain role. Regarding the country’s vigorous promotion of building energy conservation, thermal insulation materials, especially organic building insulation materials, are widely used in building insulation construction due to their excellent thermal insulation properties, low cost, pressure and water resistance. The organic thermal insulation materials currently used for outside wall insulation include polystyrene foam board (EPS), extruded polystyrene foam board (XPS), polyurethane foam board (PU), and phenolic foam board [1,2].

One of the applications of phenolic resin as insulation material is to make phenolic foam insulation board. The phenolic resin can activate the interface by the surfactant and reduce the surface tension. The foaming agent can be decomposed into gas or physically vaporized to form a honeycomb structure, and finally the phenolic foam is formed under the action of curing and curing of the curing agent. Compared with other outside wall insulation materials, the phenolic foam has many advantages in thermal insulation and fire resistance, which is known as the “king of insulation” [3,4]. Since the 1990s, phenolic composite materials, including phenolic foam, have been significantly developed. Firstly, the military of the United Kingdom and the United States has paid attention to it. It has been used in the aerospace, defense and military industries, and later used in ships, stations, oil wells and other places with strict fire protection requirements and is gradually being promoted in construction, hospitals, stadiums and other fields [5]. In recent years, phenolic foam as the sound insulation and insulation materials have been widely used in the construction industry. Japan’s Ministry of Construction has issued a decree on phenolic foams as standard building flame retardant materials, and France uses phenolic foam material as a material to seal and control fires. Many large apartments in cities such as Marseille and Lyon have phenolic foam panels installed on the walls. Ordinary phenolic resin is relatively brittle, and the phenolic hydroxyl group and methylene group in the molecular structure are easily oxidized, which limits the application of phenolic resin at high temperature. Therefore, the phenolic resin is further modified to enhance its fireproof and flame retardant properties so that a modified phenolic resin which is more suitable for the market can be obtained. Commonly used are phosphorus-based, nitrogen-based flame retardants, silicones, boron. Sang used graphene and cardanol to modify phenolic resin to get graphene and cardanol-modified phenolic resin (GCP). Filling the resin with homemade carbon fiber base paper to prepare graphene and cardanol, modified phenolic resin-based carbon fiber paper-based composite (GCPC) is created [6]. Tang and others used polyammonium phosphate (APP) as flame retardant to modify the phenolic resin composites reinforced by bamboo and polypropylene fiber composite felt. They studied the effects of adding flame retardant on the mechanical, flame retardant and thermal conductivity of the composites [7]. Li and others used borosilicate modified phenolic resin, boron and silicone to prepare a novel boron-and silicone-containing phenolic resin (BSiPF) solution, and characterized the structure, molecular weight, gel properties, curing properties and heat resistance of BSiPF. This makes introducing silicon and boron significantly improve the heat resistance and oxidation resistance of phenolic resin [8]. Wang used hydrobromic acid(HBr) and hydroiodic acid(HI) as reagents to demethylate an alkali lignin (AL) to increase its hydroxyl content and measured different thermal properties and performance of phenolic resins [9]. Yong and Yu used bio-oil as the raw material to synthesize bio-oil phenol formaldehyde resin desirable resin for fabricating phenolic-based material, which the thermal curing behavior and heat resistance of bio-oil phenol formaldehyde resins were investigated [10,11].

Melamine is a halogen-free nitrogen-based flame retardant, which has the characteristics of improving the physical and mechanical properties of products, low hygroscopicity, replacing some of the resin, reducing the amount of the adhesive. Compared to other types of flame retardants, melamine has apparent advantages. Melamine has been widely used in the modification of phenolic resins. Ge and others modified phenolic resin with melamine, and through experiments the mechanical properties of the modified phenolic foam were improved, while the free formaldehyde content decreased and the oxygen index increased. The research shows that the best melamine mass fraction is 4.5% [12]. Aluminum diethylphosphinate is a new flame retardant organic hypophosphite. It has excellent flame retardant properties, environmental and health-friendly properties, halogen-free, high flame-retardant efficiency, hydrophobic smoke suppression, and thermal stability. Now mainly used in glass fiber-reinforced polyamide 6 and 66 are composites and various polyesters (PBT and PET). Chen Lu introduced aluminum diethylphosphinate into polyethylene terephthalate (PET), and tested the flame retardancy of PET composites by vertical combustion and oxygen index. The thermal decomposition activation energy was calculated by thermo-gravimetric (TG) analyzer and the Flynn–Wall–Ozawa method, the thermal degradation behavior was further discussed [13]. Zhao employed four types of metal-mediated catalysts in the synthesis of phenolic resin resin to accelerate the curing rate and to lower the curing temperature of phenolic resin [14]. Chen and others prepared a series of polylactic acid/aluminum diethylphosphinate and polylactic acid/aluminum diethylphosphinate/organomontmorillonite composites, and analyzed them by thermo-gravimetric analysis, limiting oxygen index, and vertical combustion. The effects of aluminum diethylphosphinate and organomontmorillonite on the thermal stability and flame retardancy of polylactic acid composites were investigated by cone calorimeter. The mechanism of the synergistic flame-retardant character of aluminum diethylphosphinate and organomontmorillonite was also discussed [15].

Melamine flame retardants have been used in phenolic resins, but aluminum diethylphosphinate has not been used in phenolic resins. The two flame retardants are combined and added into phenolic resin as a new composite flame retardant. In this work, aluminum diethylphosphinate and melamine are added to the phenolic resin as excellent flame-retardant additives to improve the flame-retardant properties of phenolic resin. The nitrogen-phosphorus additive acts to make the modified phenolic resin more excellent. Through the test of its flame retardancy, thermal stability and combustion properties, the effects of the two additives on the phenolic resin were clarified, and the optimal synergistic ratio was sought, so phenolic resin could be more widely used in building insulation materials.

## 2. Materials and Methods

### 2.1. Experimental Materials

Phenolic resin, Shandong baiqian chemical Co., Ltd, Shandong, China. Aluminum diethylphosphinate, melamine, chemical pure, Sinopharm Chemical Reagent Co., Ltd, Nanjing, China. Anhydrous ethanol, Yonghua Chemical Technology (Jiangsu) Co., Ltd, Nanjing, China. All the raw materials were industrial products.

### 2.2. Preparation of Modified Phenolic Resin

Anhydrous ethanol was added to the phenolic resin, stirred to dissolve, and different ratios of aluminum diethylphosphinate and melamine were added and stirred with a constant temperature magnetic stirrer to dissolve it evenly. Then, this was subject to 30 min with an ultrasonic disperser. After standing for 20 min, the bubbles were removed, the mix was poured into a Teflon mold, and placed in a vacuum drying oven to step up the temperature. The specific addition amounts of aluminum diethylphosphinate and melamine are shown in Table 1.

### 2.3. Testing and Characterization

Fourier transform infrared spectroscopy (FTIR): measurement was carried out with IS5 infrared spectrometer (Thermo Scientific, Waltham, MA, USA); thermo-gravimetric analysis (TGA) experiment: the spline was subjected to TG test using a SDTQ600 thermo-gravimetric analyzer (TA Instruments, Lukens, DE, USA). The temperature range was 50 °C–800 °C and it was tested under a nitrogen atmosphere with a gas flow rate of 20 mL/min; limiting oxygen index (LOI) test: the standard is ISO 4589-2 [16], and we used the HC-2 type limit oxygen index instrument (Jiangning Instrument Analysis Factory, Nanjing, China) was tested, the test sample size was 100 × 10 × 4 mm^3^; vertical combustion test(UL-94): a CFZ-2 horizontal vertical burner (Jiangning Instrument Analysis Factory, Nanjing, China) was used, and the test sample size was 125 × 13 × 3 mm^3^; cone calorimeter test (CCT): this employed the ISO 5660 standard [17], and a Govmark CC-2-2128 cone calorimeter (Deatak, McHenry, IL, USA)was used to test the combustion performance of phenolic resin and its composite materials in the atmosphere of 50 kW/m^2^ heat flux. The size of the test sheet was 100 × 100 × 3 mm^3^. Scanning electron microscopy (SEM): the morphological structures of the char layer after the calorimeter test were observed by Zeiss EVO18 SEM, Oberkochen, Germany.

## 3. Results and Discussion

### 3.1. Fourier Transform Infrared Spectroscopy (FTIR) Test Results

**Figure 1:**
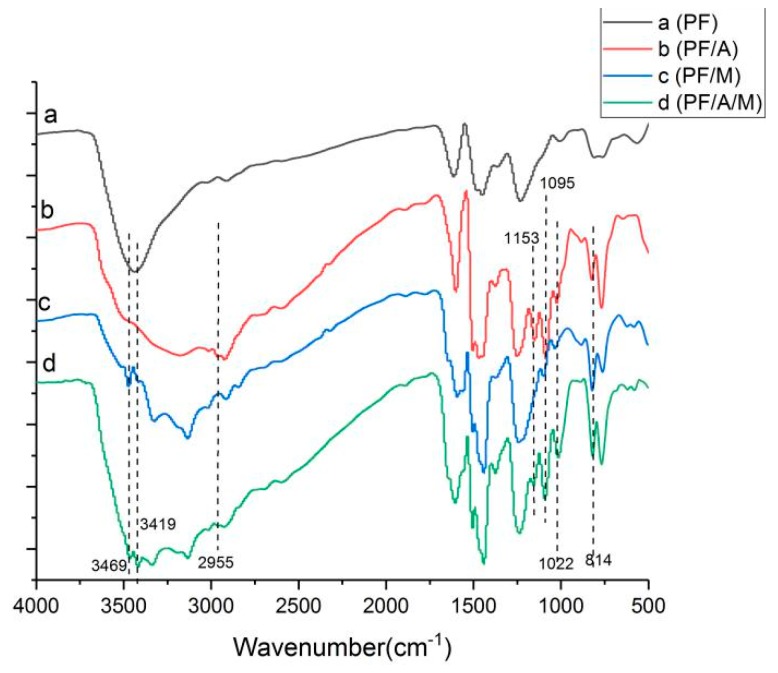
Fourier transform infrared (FTIR) spectra of phenolic resin and phenolic resin composite system.

Phenolic resin and modified phenolic resin were characterized by Fourier transform infrared spectroscopy, as shown in Figure 1. The Fourier spectra of the melamine-modified phenolic resin are shown in Figure 1, and the absorption peaks at 3469 cm^−1^ and 3419 cm^−1^ are the performance of the anti-symmetrical vibration of NH_2_. The 1022 cm^−1^ is formed by NH twisting vibration, and the characteristic absorption peak at 814 cm^−1^ is the characteristic absorption of deformation vibration of a triazine ring [18]. The absorption peaks of these bands are also found in Figure 1 of the phenolic resin system in which aluminum diethylphosphinate and melamine are simultaneously added. In Figure 1 of the aluminum diethylphosphinate modified phenolic resin system, the stretching vibration of P=C double bond is at 1153 cm^−1^, and the peak at 1095 cm^−1^ is formed by PO· symmetrical stretching vibration [19]. The characteristic peak at 2955 cm^−1^ is consistent with the stretching vibration frequency of –CH_2_– [20], and characteristic peak coincides with the characteristic peak of aluminum diethylphosphinate. The phenolic resin system with aluminum diethylphosphinate and melamine added at the same time has these absorption peak. In addition, the other characteristic peaks are consistent with the pure phenolic resin. The results were confirmed that the aluminum diethylphosphinate and melamine were synthesized successfully.

### 3.2. Thermo-Gravimetric Analysis (TGA) Results

#### 3.2.1. TGA of Modified Phenolic Resin

**Figure 2:**
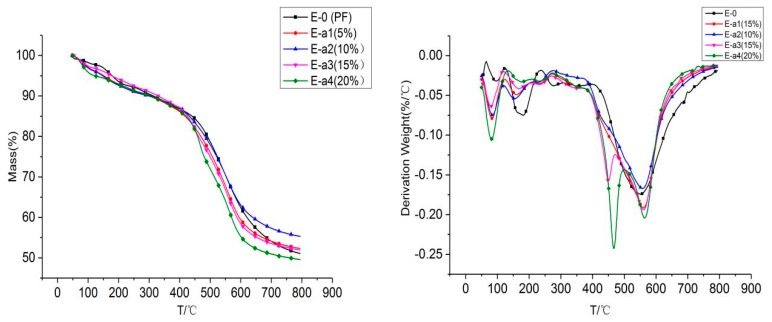
Thermo-gravimetric (TG) and derivative thermo-gravimetric (DTG) images of aluminum diethylphosphinate modified phenolic resin under nitrogen atmosphere.

**Figure 3:**
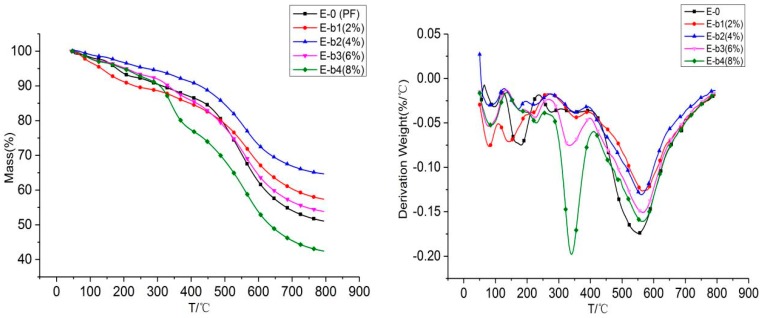
TG and DTG images of melamine modified phenolic resin under nitrogen atmosphere.

The thermal degradation performance of the modified phenolic resin was analyzed by the thermo-gravimetric test, and the melamine phenolic resin composite and the thermal degradation process under the nitrogen atmosphere. Figure 2 and Figure 3 are TGA curves of aluminum diethylphosphinate modified phenolic resin and melamine modified phenolic resin, respectively. Figure 2 is the aluminum diethylphosphinate/phenolic resin composite in a temperature range of 50 °C to 800 °C under a nitrogen atmosphere [21]. The results are shown in Table 2. T_5%_, T_10%_ and T_dmax_ in Table 2 stand for the temperature at 5 wt %, 10 wt % and maximum weight loss rate, respectively. The thermal degradation of pure phenolic resin under the nitrogen atmosphere is a one-step degradation process. When the temperature reaches 178 °C, it begins to decay. When the temperature reaches 550 °C, the thermal decomposition rate is the fastest, and carbon residue at 800 °C is 51 wt %. Compared with pure phenolic resin, the TGA curves of aluminum diethylphosphinate/phenolic resin composites are different. When the addition amount reaches 15%, two degradation processes occur at 465 °C and 560 °C, respectively. The T_dmax_ of E-0, E-a1, E-a2, E-a3 and E-a4 are 550, 562, 557, 561 and 467 °C respectively. The maximum thermal weight loss rates for E-0, E-a1, E-a2, E-a3 and E-a4 are 0.174, 0.191, 0.167, 0.194 and 0.203%/°C. The thermal stability of aluminum diethylphosphinate/phenolic resin composites is significantly different compared with pure phenolic resin. The figure shows that when the added amount of aluminum diethylphosphinate is about 10%, residual carbon of the composite at 800 °C is the largest, which is 55 wt %. When the amount of aluminum diethylphosphinate is increased to 20%, the residual carbon rate is reduced, the maximum mass loss rate becomes larger. This is because aluminum diethylphosphinate acts as a flame retardant through the gas phase flame retardant mechanism, and when the temperature rises, aluminum diethylphosphinate degrades the phosphorus-containing compound. During combustion, it is decomposed into diethylphosphonic acid, which volatilizes to the gas phase and inhibits combustion by capturing free radicals produced by combustion [22]. However, when added in a large amount, aluminum diethylphosphinate will reduce the thermal stability of phenolic resin. Excessive aluminum diethylphosphinate addition may affect the molecular properties of the phenolic resin, thus reducing the flame retardancy and thermal stability of the phenolic resin. Therefore, the aluminum diethylphosphinate should be in the range of 10%~15%, which cannot only ensure thermal stability, but also perform its flame-retardant carbon properties.

Figure 3 is the TGA curve of phenolic resin and melamine phenolic resin composite material in the temperature range of 50–800 under the nitrogen atmosphere [23]. The results are shown in Table 3. T_5%_, T _10%_ and T_dmax_ in Table 3 stand for the temperature at 5 wt %, 10 wt % and maximum weight loss rate, respectively. The TGA curve trend of E-b1, E-b2, E-b3 and E-b4 samples is similar to that of pure phenolic resin, which is mainly composed of two thermogravimetry stages. From Figure 3, we can see that the thermal degradation of pure phenolic resin under the nitrogen atmosphere is a one-step degradation process. When the temperature reaches 178 °C, it begins to decay. When the temperature reaches 550 °C, the thermal weight loss rate of pure phenolic resin reached the maximum at 550 °C and is 0.174 %/°C. By adding melamine into phenolic resin, the curves are different. The maximum thermal weight loss rates corresponding to E-b1, E-b2 and E-b3 are 0.126, 0.13 and 0.151%/°C, which are all lower than 0.174 %/°C of the E-0 groups. When the addition of 8% melamine to phenolic resin, the thermal weight loss rate of E-b4 is different to the other group, and its maximum thermal weight loss rate is 0.197%/°C at 340 °C. A peak appeared between 270 °C to 410 °C, and the peak value increased with the increase of the amount of melamine added. The decomposition stage at this stage is due to the decomposition of melamine. The second thermal weight loss phase occurs between 410 °C –650 °C, mainly due to the fracture of the phenolic resin main chain caused. At this stage, more obvious peaks can be seen from the DTG curve. This is because with the addition of melamine, the thermal weight loss rate of the composite gradually decreases. The melamine phenolic resin composite has a T_10%_ ratio of 231 °C, 426 °C, 331 °C and 306 °C, and the residual carbon content at 800 °C is 57%, 64%, 53%, 42%. Thus it can be seen that with the addition of 4% melamine to phenolic resin, the residual carbon content reached the maximum, and the maximum mass loss rate was also small. This shows that the thermal stability of the melamine phenolic resin composite was better at 4% addition, and there was residual in the synthesis of phenolic resin. There is residual formaldehyde in the synthesis of phenolic resin, and formaldehyde is flammable. With the addition of melamine, it reacts with formaldehyde to form melamine formaldehyde, which consumes a part of residual formaldehyde. Moreover, the generated melamine formaldehyde contains nitrogen rings, which have a good flame retardant effect [24], thus increasing the thermal stability of phenolic resin.

#### 3.2.2. TGA of Phenolic Resin Modified by Synergistic Modification of Aluminum Diethylphosphinate and Melamine

**Figure 4:**
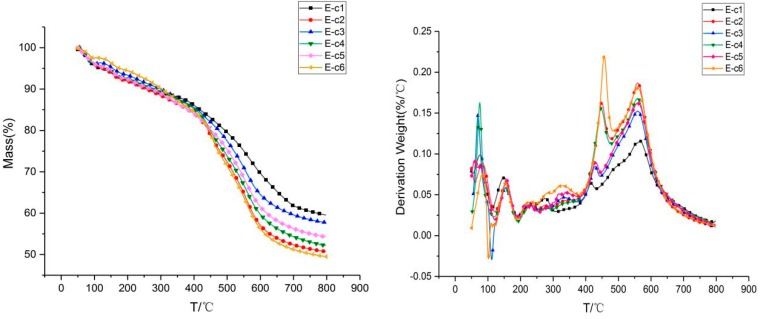
TG and DTG images of aluminum diethylphosphinate and melamine modified phenolic resin under nitrogen atmosphere.

The thermo-gravimetric analysis curve was used to study the thermal stability of the aluminum diethylphosphinate and melamine synergistic modified phenolic resin composites and the thermal degradation process. Figure 4 is the TGA curve of a phenolic resin and its composite material in a temperature range of 50 °C to 800 °C under a nitrogen atmosphere. Some results are shown in Table 4. T_5%_, T_10%_ and T_dmax_ in Table 4 stand for the temperature at 5 wt %, 10 wt % and maximum weight loss rate, respectively. According to Figure 3, the TG curves of the six groups of E-c1, E-c2, E-c3, E-c4, E-c5 and E-b6 have similar trends, mainly composed of two thermo-gravimetric stages. The T_dmax_ values of the phenolic resin composite system were 570 °C, 560 °C, 559 °C, 559 °C, 559 °C, and 561 °C, respectively. Under the same range of temperatures, the residual carbon content at 800 °C was 59.5%, 50.7%, 57.7%, and 52.2%, 54.3%, 49.5%. When adding 3 wt % melamine and 10 wt % aluminum diethylphosphinate, the residual carbon amount is up to 59.5%, adding 10 wt % aluminum diethylphosphinate alone, the residual carbon amount is 55%. The carbon residue rose when the melamine was added to phenolic resin, which means that melamine can greatly improve the thermodynamic stability of phenolic resin. When the amount of aluminum diethylphosphinate and melamine added are 15% and 5% by weight, the residual carbon is 49.5%. Compared with the pure phenolic resin, the stability has not been improved because the flame retardant is excessive, which causes the thermodynamic properties of the phenolic resin to be negatively affected.

### 3.3. Modified Phenolic Resin Flame-Retardant Properties

The flame-retardant properties of different resin systems were tested by LOI and the UL-94 vertical burning test [25]. Table 5 and Table 6 presents the LOI values and UL-94 test results of the samples.

According to Table 5, we can seen that the LOI value of pure phenolic resin is 30.0%, which is inherently flame-retardant, and almost no dripping is formed in the vertical burning test. Compared with that of pure phenolic resin, the LOI was 33.1% when the amount addition reached 10% by weight, and passed the UL-94 V-0 level in the vertical burning test. Continuing to increase the amount of flame retardant, when the addition amount reaches 15 wt%, the LOI of phenolic resin composites is 34.6%, and in the vertical burning test, can pass the UL-94 V-0 level. However, when the aluminum diethylphosphinate was 20 wt %, the flame-retardant capacity of phenolic resin decreased with the increase of the aluminum diethylphosphinate. It can be seen that when the addition amount of aluminum diethylphosphinate is 15%, the LOI and UL-94 test results of the phenolic resin composite system are the best, which can improve the flame-retardant performance of the phenolic resin. This highlights that most of the decomposition products of aluminum diethylphosphinate are volatilized into the gas phase during combustion, and may produce free radicals such as PO· in the gas phase, and capture free radicals produced by combustion to inhibit combustion [26,27]. When melamine was added to the phenolic resin, the LOI increased first and then decreased with the addition amount. When the addition reached 4 wt %, the LOI reached a maximum of 32.1%. In the vertical burning test, it can pass the UL-94 V-0 level. Then, with the increase of the amount of addition, the LOI began to decrease, showing that adding a suitable amount of melamine can improve the flame-retardant properties of the phenolic resin.

The flame-retardant properties of aluminum diethylphosphinate and melamine were simultaneously added to the phenolic resin, as shown in Table 6. When the added amount of aluminum diethylphosphinate is 10 wt % and melamine is 4 wt %, the LOI of the phenolic resin composite system reaches a maximum value of 35.8%, and can pass the UL-94 V-0 grade. Compared with adding melamine alone, there is a significant improvement. With the addition of aluminum diethylphosphinate into phenolic resin, the phosphorus-containing group is thermally oxidized and decomposed during combustion to form phosphoric acid-promoting charcoal, suggesting that aluminum diethylphosphinate and melamine can play a synergistic role in the phenolic resin composite system. The synergistic effect of the nitrogen-phosphorus ion improves the flame retardancy. When the added amount of the two flame retardants is increased, the LOI value is decreased, indicating the addition amount has exceeded the appropriate range. If too much flame retardant is added, the flame retardancy of the phenolic resin system is lowered.

### 3.4. Cone Calorimeter Test (CCT) of Modified Phenolic Resin

The cone calorimeter test (CCT) can test the combustion properties of composites under real fire conditions, and it is one of the most important tests to study the flame retardant properties of composites [28]. The matching results of CCT were summarized in Table 7, including peak heat release rate (p_HRR_), time corresponding to pHRR (T_pHRR_), time to sustained ignition (TTI), total heat release (THR) and amount of carbon residue [29].

**Figure 5:**
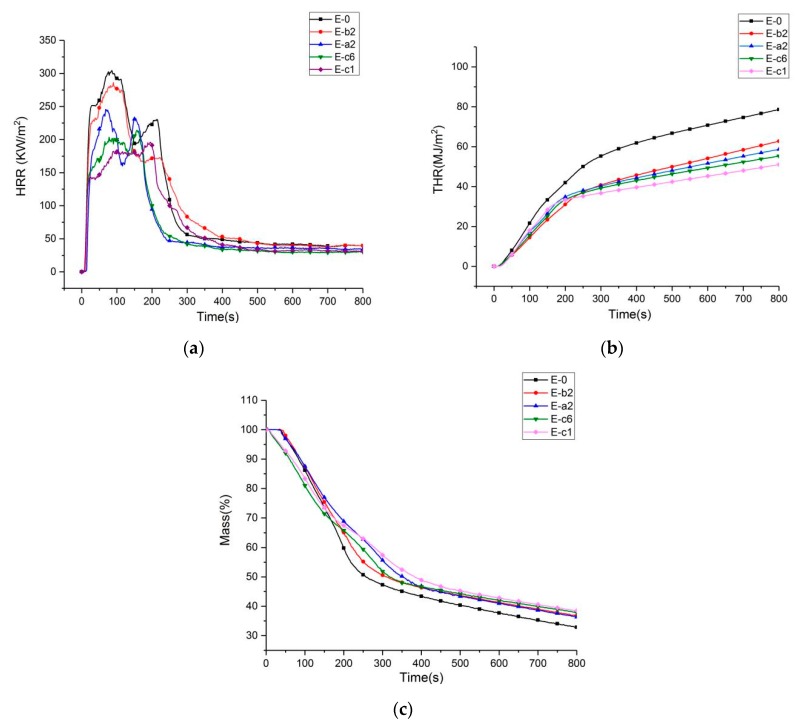
Heat release rate (**a**), total heat release (**b**) and weight loss curve (**c**) of phenolic resin and its composites.

The HRR, THR, and mass loss plots of phenolic resin and its composites during combustion are shown in Figure 5. As can be seen from Figure 5 and Table 7, the HRR of pure phenolic resin increases sharply after ignition and reaches a peak heat release rate of 304.4 kW/m^2^ at 86 s, and THR was as high as 78.6 MJ/m^2^, TTI was 26 s, and the carbon residue was 32.8%. This shows that pure phenolic resin has a high risk of burning. When 10 wt % aluminum diethylphosphinate was added to the phenolic resin, the HRR of the E-a2 group decreased to 245.6 kW/m^2^, the time was extended to 104 s, and the THR decreased to 58.6 MJ/m^2^. Compared with pure phenolic resin, HRR and THR decreased by 19.3% and 25.4%, respectively. TTI increased to 34 s, and increased by 8 s compared with pure phenolic resin. After adding 10 wt % aluminum diethylphosphinate and 3 wt % melamine to the phenolic resin, the HRR of the E-c1 group decreased to 196.2 kW/m^2^ and the THR decreased to 51 MJ/m^2^. Compared with the pure phenolic resin, the HRR decreased by 35.5% and 35.1%, respectively. The TTI increased to 42, and the pure phenolic resin increased by 16 s. From the mass loss image of Figure 5c, it can be seen clearly that the mass loss curve of E-c1 is much more backward than pure phenolic resin. Compared with the pure phenolic resin residual carbon content of 32.2 wt %, the residual carbon content of E-c1 group is increased by 19.6%, which is 38.5 wt %. The simultaneous addition of aluminum diethylphosphinate and melamine is more effective than the phenolic resin system with only aluminum diethylphosphinate added. The simultaneous action of the two flame retardants can effectively improve the stability of the phenolic resin during combustion, reduce the heat release rate, heat released and the amount of residual carbon increase. Aluminum diethylphosphinate mainly produces flame retardant effect through the gas phase, while melamine can increase the amount of residual carbon and reduce the heat release rate.

By adding 4 wt % melamine to the phenolic resin, the HRR of the E-b2 group decreased to 286.6 kW/m^2^, and the time was extended to 90 s. Compared with pure phenolic resin, HRR and THR decreased by 5.8% and 20.2%, respectively. The TTI increased to 29 s. Compared with the phenolic resin system with aluminum diethylphosphinate and melamine added at the same time, the thermal stability was obviously reduced when burning. When 15 wt % aluminum diethylphosphinate and 5 wt % melamine were added to the phenolic resin, the HRR reached a maximum of 213.9 kW/m^2^ at 158 s, and THR values could reach 55.3 MJ/m^2^. Compared with pure phenolic resin, the pHRR and THR of E-c6 decreased by 29.7% and 29.6%, respectively. It can be seen that compared with the E-c1 group phenolic composite material, the combustion performance of the E-c6 composite material was not good, which means that the excessive flame-retardant combustion performance is not effective.

**Figure 6:**
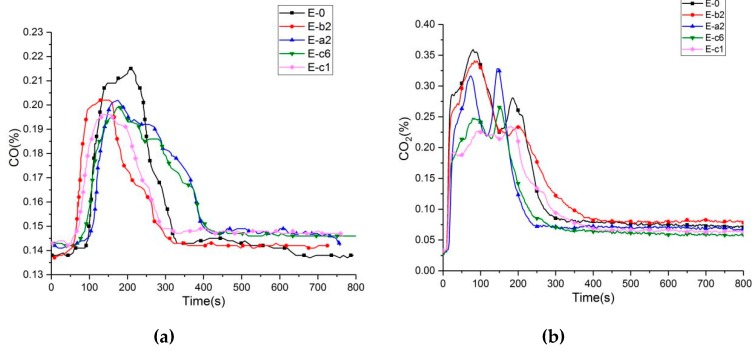
CO (**a**) and CO_2_ (**b**) curves of phenolic resin and its composites.

According to research on the causes of death of those killed in fires, about two-thirds of deaths are caused by the direct poisoning of CO generated in the fire. Although CO_2_ is non-toxic, when it reaches a certain concentration, it will also cause harm to the human body. Therefore, it is particularly important to reduce the content of CO and CO_2._ Figure 6 shows CO and CO_2_ curves of all composites; the results are summarized in Table 8, including peak concentration of CO (p_CO_) and CO_2_ (p_CO2_), pink time of CO(Tp_CO_) and CO_2_ (Tp_CO2_). The CO of pure phenolic resin increased sharply after ignition and reached the peak of 0.215% at 206 s, and CO_2_ concentration was as high as 0.356% at 82 s. When 10 wt % aluminum diethylphosphinate was added to the phenolic resin, the maximum concentration of CO and CO_2_ of the E-a2 group decreased to 0.201% at 152 s and 0.341%, respectively. Compared with pure phenolic resin, they decreased by 6.5% and 9%, respectively. By adding 10 wt % aluminum diethylphosphinate and 3 wt % melamine to the phenolic resin, the peak concentration of CO and CO_2_ of the E-c1 group decreased to 0.196% and 0.222%, respectively. Compared with the pure phenolic resin, the content decreased by 8.8% and 37.6%, respectively. With the addition of 15 wt % aluminum diethylphosphinate and 5 wt % melamine to the phenolic resin, the concentration of CO and CO_2_ of the E-c1 group decreased to 0.199% and 0.264%, respectively. Compared with the pure phenolic resin, they decreased by 7.4% and 0.134%, respectively. It is obvious that the CO and CO_2_ concentrations were the minimum by adding 10 wt % aluminum diethylphosphinate to the phenolic resin, the hazard of the phenolic resin can be reduced.

### 3.5. Analysis of Char Residual

**Figure 7:**
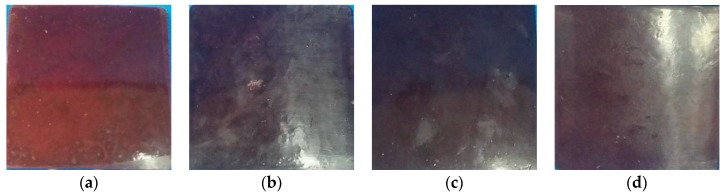
Digital photographs of pre-CCT samples of E-0 (**a**), E-a2 (**b**), E-b2 (**c**) and E-c1 (**d**).

**Figure 8:**
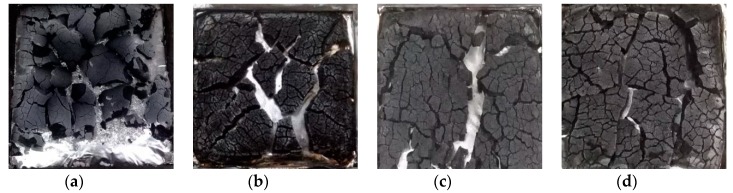
Digital photographs of CCT samples after E-0 (**a**), E-a2 (**b**), E-b2 (**c**) and E-c1 (**d**).

To further understand the flame-retardant effect of the modified phenolic resin, the residual char after the cone calorimeter test was analyzed. Figure 7 and Figure 8 are digital photographs of phenolic resin and composite materials before and after combustion under the same thermal radiation conditions in a cone calorimeter test.

As can be seen from the figures, pure phenolic resin is completely burned, and only a few carbon residues were left, and the residual carbon is obviously loose, light and thin. However, the carbon layer of E-a2 and E-b2 composites has a significant degree of expansion and an increase in the amount of carbon residue, as shown in Table 9. The thicknesses of the residual carbon layers in the E-0, E-a2, E-b2 and E-c1 groups were 2.5, 5.5, 5 and 5.8 mm, respectively. Compared with pure phenolic resin, the thickness of the residual carbon layer increased significantly. When aluminum diethylphosphinate and melamine are added together, the carbon residue of E-c1 is significantly increased compared to pure phenolic resin. The results show that the nitrogen-phosphorus ion obviously increases the amount of carbon residue and the strength of carbon layer after phenolic resin combustion.

**Figure 9:**
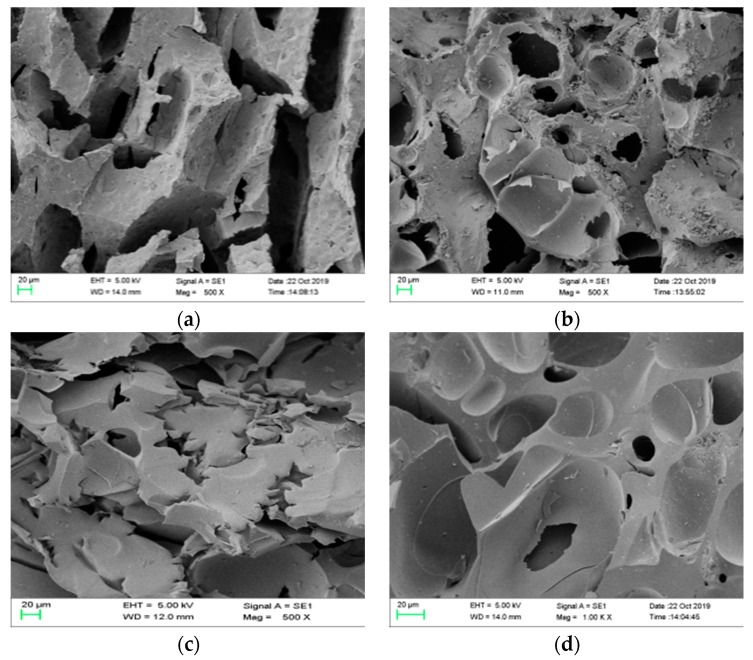
Electron micrograph of carbon layer after CCT test of phenolic resin and phenolic resin composites: E-0 (**a**), E-a2 (**b**), E-b2 (**c**) and E-c1 (**d**).

In order to explore the microstructure of the carbon layer of the phenolic composite, the carbon layer after the combustion of the pure phenolic resin and phenolic resin composites was analyzed by SEM. It can be observed from Figure 9a that the pure phenolic resin had a relatively porous hole and a large crack on the surface of the carbon layer after the CCT test, which is attributed to the rapid volatilization of the flammable volatile gas produced by the combustion on the pure phenolic resin surface. With the addition of aluminum diethylphosphinate, as shown in Figure 9b, the carbon layer of the phenolic resin composite system still had large pores, and the carbon layer was rather loose, which did not block oxygen and heat. This is because aluminum diethylphosphinate generates volatile diethyl hypophosphorous acid during degradation, which acts as a trapping free radical in the gas phase and blocks the combustion process. With the volatilization of diethyl hypophosphorous acid, the phosphorus content inside the phenolic resin substrate decreases rapidly, resulting in a decrease in the char forming ability of the system. Therefore, the effect of the condensed phase flame-retardant mechanism is not obvious [30], which can be explained through TGA. Therefore, the final residual amount of phenolic composite system with aluminum diethylphosphinate at 800 °C in TGA analysis has a small increase compared with that of pure phenolic resin material. When the phenolic system was introduced into melamine, as shown in Figure 9c, the carbon layer of the phenolic composite material did not have pores and cracks such as pure phenolic resin, and became smoother and finer. This is because melamine reacts with formaldehyde after it is added. The formation of melamine formaldehyde and melamine formaldehyde disturbs the intermolecular rigid structure of phenolic resin, and forms an irregular cross-linked interpenetrating network structure with phenolic resin, which hinders the decomposition of phenolic resin and needs higher energy in decomposition [31]. It can be explained that in the TGA analysis the final carbon residue of the phenolic composite system with melamine introduced at 800 °C was higher than that of the pure phenolic resin material. The carbon layer of the phenolic system with aluminum diethylphosphinate and melamine added at the same time, as shown in Figure 9d, was more compact, continuous and dense, and there were almost no large cracks and holes. This shows that the combined action of the two can play a good role in heat insulation and oxygen insulation, and can slow down the mass and heat transfer and the release of combustible volatiles, so as to prevent the further combustion of phenolic resin composite materials.

**Figure 10:**
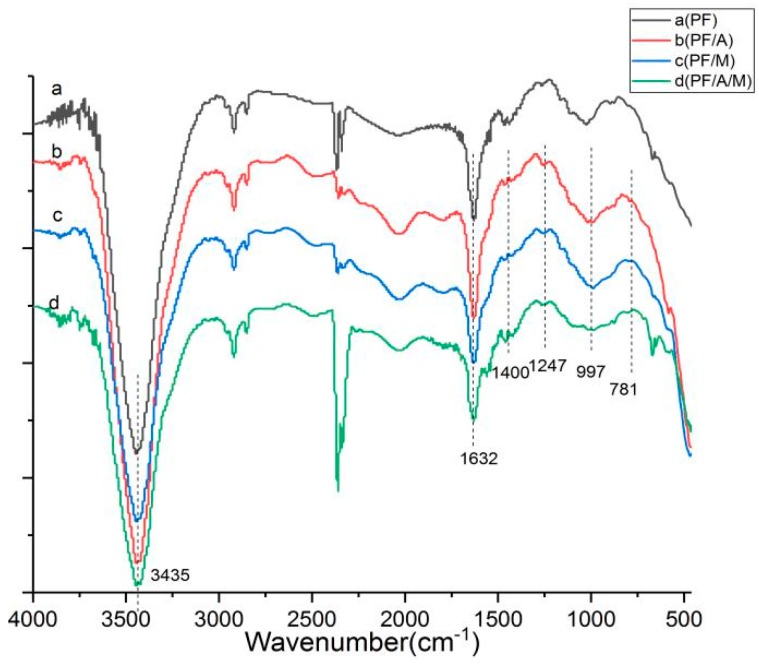
FTIR spectra of phenolic resin and phenolic resin composite system after the cone calorimeter test.

The FTIR spectra of the char residues after the cone calorimeter test are shown in Figure 10. By comparing with the spectra of the Figure 1, it can be seen that the peaks at 3469 cm^−1^ and 3419 cm^−1^ (NH_2_ groups), 2955 cm^−1^ (–CH_2_ groups), 1153 cm^−1^ (P=C groups) disappear, and some new peaks are formed during combustion, as shown in Figure 10, indicating that the cross-linking reaction occurred between the phenolic resin and two flame retardants. The strong characteristic peaks in the range of 3500–3100 cm^−1^ are attributed to the stretching vibration of –OH and –NH– groups. The peaks at 1632, 1400 and 997 cm^−1^ are due to the C=C absorption in carbonization reaction, P-N groups and P–O–C groups, respectively. After the addition of aluminum diethylphosphinate, a new peak is observed at 1247 cm^−1^ (P=O groups) and 781 cm^−1^ (Al–O groups) in Figure 10. Additionally, the char rich in P–O–P groups, P-O-C groups, P-N groups and C=C groups exhibits a more intumescent and compact structure, which gives an illustration of the lower heat release and gas emissions during the combustion process.

## 4. Conclusions

In this work, various concentrations of aluminum diethylphosphinate and melamine were successfully added to the phenolic resin to synthesize the modified phenolic resin. Through thermo-gravimetric analysis and the test of flame retardancy, the suitable addition amount of the two flame retardants was obtained. The results show that when 15 wt % of aluminum diethylphosphinate is added to the phenolic resin, the flame retardancy of the modified resin can pass the UL-94 V-0 flame-retardant grade, the LOI reaches 34.6%, and the residual carbon content is 52%. With the addition of 4 wt % melamine, the flame retardancy of the modified resin can pass the UL-94 V-0 flame retardant grade, the LOI reaches 32.1%, and the residual carbon amount is 64%. It can be seen that aluminum diethylphosphinate has a significant effect in improving the flame retardancy of the phenolic resin, and can increase LOI of the phenolic resin to 34.6%, while melamine can improve the thermal stability of phenolic resin, improve the carbon residue and reduce the rate of thermal decomposition. The test results of the cone calorimeter also show that when the aluminum diethylphosphinate and melamine are added together in the phenolic resin, the combustion performance is better than the addition of one of the flame retardants alone. With the addition of 10 wt% aluminum diethylphosphinate and 3 wt % melamine into the phenolic resin, the flame retardancy of the modified resin can pass the UL-94 V-0 flame-retardant grade, and the LOI reaches 34.6%. The residual carbon content increases to 59.5%, and the HRR decreases to 196.2 kW/m^2^ at 196 s, which is relatively pure phenolic resin decreased by 35.5%, THR decreased to 51 MJ/m^2^ and TTI increased to 42 s. The peak content of CO and CO_2_ decreased to 0.196% and 0.222%, compared with the pure phenolic resin, and the content decreased by 8.8% and 37.6%, respectively. In the SEM experiment, the electron micrograph of residual carbon also verified that the carbon layer of the phenolic system with aluminum diethylphosphinate and melamine added at the same time is more compact, continuous and dense, while there are almost no large cracks and holes, indicating that the two flame retardants can work well together. It acts as a heat insulator and oxygen barrier to slow the release of mass and heat transfer and flammable volatiles, thereby preventing further combustion of the phenolic resin composite. All the above suggest that aluminum diethylphosphinate and melamine play a nitrogen-phosphorus synergistic effect in the phenolic resin, which improves the thermal stability and flame retardancy of the phenolic resin. The thermal stability and flame retardancy of phenolic resin obtained can be used in further tests on pyrolysis and combustion in building materials.

## Figures and Tables

**Table 1:** Samples of phenolic resin composite materials containing different additives.

Sample Codes	Phenolic Resin (wt %)	Aluminum Diethylphosphinate (wt %)	Melamine (wt %)
E-0	100	0	0
E-a1	95	5	0
E-a2	90	10	0
E-a3	85	15	0
E-a4	80	20	0
E-b1	98	0	2
E-b2	96	0	4
E-b3	94	0	6
E-b4	92	0	8
E-c1	87	10	3
E-c2	82	15	3
E-c3	86	10	4
E-c4	81	15	4
E-c5	85	10	5
E-c6	80	15	5

**Table 2:** TG data of phenolic resin and its composites.

Material	T_5%_ (°C)	T_10%_ (°C)	T_dmax_ (°C)	Char Residues at 800 °C (wt%)
E-0	178	313	550	51
E-a1	151	300	562	52
E-a2	147	299	557	55
E-a3	177	329	561	52
E-a4	121	305	467	49

**Table 3:** TG data of phenolic resin and its composites.

Material	T_5%_ (°C)	T_10%_ (°C)	T_dmax_ (°C)	Char Residues at 800 °C (wt %)
E-0	178	313	550	51
E-b1	133	231	559	57
E-b2	268	426	560	64
E-b3	206	331	566	53
E-b4	198	306	340	42

**Table 4:** TG data of phenolic resin composites.

Material	T_5%_ (°C)	T_10%_ (°C)	T_dmax_ (°C)	Char Residues at 800 °C (wt %)
E-c1	121	265	570	59.5
E-c2	125	256	560	50.7
E-c3	155	299	559	57.7
E-c4	137	281	559	52.2
E-c5	138	271	559	54.3
E-c6	176	303	561	49.5

**Table 5:** Limiting oxygen index (LOI) and vertical burning test (UL-94) results of phenolic resin and its composites.

Sample Codes	Flame Retardants Mass Fraction(%)	LOI	UL-94
**Aluminum Diethylphosphinate**			
E-0	0	30.0	V-2
E-a1	5	31.0	V-1
E-a2	10	33.1	V-0
E-a3	15	34.6	V-0
E-a4	20	32.3	V-0
**Melamine**			
E-b1	2	30.8	V-1
E-b2	4	32.1	V-0
E-b3	6	31.6	V-0
E-b4	8	30.4	V-1

**Table 6:** LOI and UL-94 results of phenolic resin composites.

Sample Codes	Aluminum Diethylphosphinate(wt %)	Melamine (wt %)	LOI	UL-94
E-c1	10	3	34.6	V-0
E-c2	15	3	34.8	V-0
E-c3	10	4	35.8	V-0
E-c4	15	4	34.4	V-0
E-c5	10	5	34.1	V-0
E-c6	15	5	34.0	V-0

**Table 7:** Cone calorimeter test (CCT) data of phenolic resin and its composites.

Sample Codes	TTI (s)	p_HRR_ (kW/m^2^)	T_PHRR_ (s)	THR (MJ/m^2^)	Residues (wt %)
E-0	26	304.4	86	78.6	32.2
E-b2	29	286.6	90	62.7	36.7
E-a2	34	245.6	104	58.6	36.3
E-c6	38	213.9	158	55.3	37.9
E-c1	42	196.2	196	51	38.5

**Table 8:** Gas data of phenolic resin and its composites.

Sample Codes	p_CO_ (%)	Tp_CO_ (s)	p_CO2_ (%)	Tp_CO2_ (s)
E-0	0.215	206	0.356	82
E-b2	0.202	152	0.341	90
E-a2	0.201	175	0.324	150
E-c6	0.199	183	0.264	154
E-c1	0.196	160	0.222	138

**Table 9:** Thickness of residual carbon layer.

Sample Codes	Thickness of Residual Carbon Layer (mm)
E-0	2.5
E-a2	5.5
E-b2	5
E-c1	5.8
